# Long-read transcriptome and other genomic resources for the angiosperm *Silene noctiflora*

**DOI:** 10.1093/g3journal/jkab189

**Published:** 2021-06-03

**Authors:** Alissa M Williams, Michael W Itgen, Amanda K Broz, Olivia G Carter, Daniel B Sloan

**Affiliations:** 1 Department of Biology, Colorado State University, Fort Collins, CO 80523, USA; 2 Cell and Molecular Biology Graduate Program, Colorado State University, Fort Collins, CO 80523, USA

**Keywords:** *Silene*, PacBio, Iso-Seq, transcriptome, *Silene noctiflora*, karyotype, genome assembly

## Abstract

The angiosperm genus *Silene* is a model system for several traits of ecological and evolutionary significance in plants, including breeding system and sex chromosome evolution, host-pathogen interactions, invasive species biology, heavy metal tolerance, and cytonuclear interactions. Despite its importance, genomic resources for this large genus of approximately 850 species are scarce, with only one published whole-genome sequence (from the dioecious species *Silene latifolia*). Here, we provide genomic and transcriptomic resources for a hermaphroditic representative of this genus (*S. noctiflora*), including a PacBio Iso-Seq transcriptome, which uses long-read, single-molecule sequencing technology to analyze full-length mRNA transcripts. Using these data, we have assembled and annotated high-quality full-length cDNA sequences for approximately 14,126 *S. noctiflora* genes and 25,317 isoforms. We demonstrated the utility of these data to distinguish between recent and highly similar gene duplicates by identifying novel paralogous genes in an essential protease complex. Furthermore, we provide a draft assembly for the approximately 2.7-Gb genome of this species, which is near the upper range of genome-size values reported for diploids in this genus and threefold larger than the 0.9-Gb genome of *Silene conica*, another species in the same subgenus. Karyotyping confirmed that *S. noctiflora* is a diploid, indicating that its large genome size is not due to polyploidization. These resources should facilitate further study and development of this genus as a model in plant ecology and evolution.

## Introduction

*Silene* is the largest genus in the angiosperm family Caryophyllaceae and serves as a model system in many fields of ecology and evolutionary biology (Bernasconi *et al.* 2009; Jafari *et al.* 2020). For instance, *Silene* is used to study breeding system evolution, as the genus includes hermaphroditic, gynodioecious, gynomonoecious, monoecious, and dioecious species (Desfeux *et al.* 1996; Charlesworth 2006). Despite the diversity of *Silene* sexual systems, there is only one available whole-genome sequence for the entire genus—from the dioecious species *Silene latifolia*, which has heteromorphic XY sex chromosomes (Papadopulos *et al.* 2015; Krasovec *et al.* 2018). Whole-genome resources are not available for any of the hermaphroditic species, which has limited comparative genomic studies into the evolution of dioecy within this genus.

*Silene* is also used as a model system for investigating organelle genome evolution and the coevolution between nuclear and cytoplasmic genomes (*i.e.*, cytonuclear interactions) (Olson and Mccauley 2002; Städler and Delph 2002; Klaas and Olson 2006; Garraud *et al.* 2011). *Silene conica* and *S. noctiflora* have two of the largest known plant mitochondrial genomes at 11 and 7 Mb, respectively (Sloan *et al.* 2012a). In contrast, the mitochondrial genome of *S. latifolia* is only 0.25 Mb, about 45 times smaller than that of *S. conica* (Sloan *et al.* 2012a). Interestingly, the *Silene* species with expanded mitogenomes also display unusually high evolutionary rates and structural changes in mitochondrial and plastid DNA (Mower *et al.* 2007; Sloan *et al.* 2012a). The natural variation in organelle genome evolution found in this genus has been used to study how these differences affect cytonuclear interactions (Havird *et al.* 2015; Williams *et al.* 2019).

The ability to use *Silene* as a model for cytonuclear evolution is still limited by the lack of extensive nuclear genome resources. Previous work has characterized *Silene* nuclear genome size and chromosome number. Nuclear genome sizes in the genus vary considerably, although not as starkly as mitochondrial genome sizes, ranging roughly 4.5-fold among diploids (haploid sizes of 0.71 to 3.23 Gb) and eightfold when the tetraploid *S. stellata* (5.77 Gb) is included (Kruckeberg 1960; Siroký *et al.* 2001; Bai *et al.* 2012; Dagher-Kharrat *et al.* 2013; Pellicer and Leitch 2020). Most of the available nuclear sequence data come from short-read RNA sequencing, which has been conducted on multiple *Silene* species (Blavet *et al.* 2011; Sloan *et al.* 2012b; Muyle *et al.* 2012; Casimiro-Soriguer *et al.* 2016; Havird *et al.* 2017; Bertrand *et al.* 2018; Balounova *et al.* 2019). These datasets have provided an important resource for molecular studies of *Silene*, but are limited because of the challenges associated with assembling short-read sequences, especially in distinguishing similar sequences arising from gene duplication, heterozygosity, and/or alternative splicing (Alkan *et al.* 2011; Schatz *et al.* 2012; Hahn *et al.* 2014; Lan *et al.* 2017).

We have generated genomic resources critical for investigations into *S. noctiflora*, a species of interest due to its extremely unusual organelle evolution and resultant use as a model for cytonuclear interactions, as well as its status as a hermaphrodite in a genus representing many types of breeding system. We include a high-quality transcriptome using long-read PacBio Iso-Seq technology, genome size estimates, and a draft nuclear genome assembly. These resources will expand opportunities for molecular and ecological studies within the genus.

## Materials and methods

### Study system

**Figure 1:**
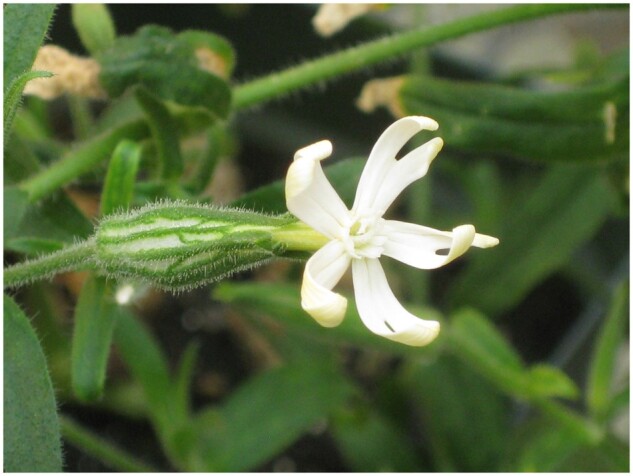
*Silene noctiflora*, also known as the night-flowering catchfly.

*Silene noctiflora* (Figure 1) is largely hermaphroditic but can produce a mixture of hermaphroditic and male-sterile flowers on the same plant (gynomonoecy) (Davis and Delph 2005). Also known as the night-flowering catchfly, this annual species is native to Eurasia and introduced throughout much of the world (McNeill 1980; Davis and Delph 2005).

### Plant growth conditions, tissue sampling, and nucleic acid extractions

Plants used for genome sequencing, Iso-Seq, and flow cytometry estimates of genome size were grown under standard greenhouse conditions with 16-hour light/8-hour dark at Colorado State University (Table 1). DNA for short-insert paired-end Illumina libraries was extracted from leaf tissue of a 7-week-old *S. noctiflora* individual from an Opole, Poland (OPL) population using a Qiagen Plant DNeasy kit. To obtain sufficient DNA quantity for construction of Illumina mate-par libraries, additional DNA was extracted from the same individual 6 weeks later using a modified CTAB protocol (Doyle and Doyle 1987) for construction of Illumina mate-pair libraries. For Iso-Seq library construction, RNA was extracted from a single 12-week-old *S. noctiflora* OPL individual (grown from seed of the plant used for DNA extraction), using a Qiagen Plant RNeasy kit. RNA extractions were performed for four different tissue samples: (1) a large flower bud with calyx removed, (2) an entire smaller flower bud including calyx, (3) the most recent (top-most) pair of cauline leaves, and (4) one leaf from the second most recent pair of cauline leaves. The four RNA extractions were quantified with Qubit RNA BR kit (Thermo Fisher Scientific). Purity and integrity were assessed with a NanoDrop 2000 (Thermo Fisher Scientific) and TapeStation 2200 (Agilent Technologies). Different tissues and developmental stages were sampled (and eventually pooled; see below) to capture a larger diversity of transcripts and thereby increase the number of genes represented.

**Table 1:** Genome sizes determined by flow cytometry

				Mean genome size	
Species	Population	Location	Samples, 2C (pg)	2C (pg)	1C (Gb)
*Silene noctiflora*	OPL^a^	Opole, Poland	5.65, 5.61, 5.46, 5.44	5.54	2.71
	OSR	Giles County, VA	5.75, 5.61	5.68	2.78
	BRP	Nelson County, VA	5.63, 5.57	5.60	2.74
*Silene conica*	ABR	Abruzzo, Italy	1.92, 1.92, 1.88	1.91	0.93
*Silene vulgaris*	S9L	Giles County, VA	2.19, 2.16	2.18	1.07
*Silene latifolia*	UK2600	Bedford County, VA	5.46, 5.45	5.46	2.67

a The *S. noctiflora* OPL population was used for Iso-Seq, genome assembly, and karyotyping.

Units: pg, picogram; Gb, gigabase; 1C, haploid amount; 2C, diploid amount.

### PacBio Iso-Seq transcriptome sequencing and analysis

Iso-Seq is an application of Pacific Biosciences (PacBio) long-read sequencing technology that uses cDNA templates to generate high quality reads for full-length transcripts. The high error rate generally associated with PacBio sequencing is drastically reduced using circular consensus sequencing (CCS), which uses hairpin adapters on each end of a double-stranded molecule to create a circular, single-stranded topology (Au *et al.* 2012; Rhoads and Au 2015; Hestand *et al.* 2016; Wenger *et al.* 2019). This topology allows the polymerase to read the same full-length molecule multiple times over, generating an accurate consensus sequence (Ono *et al.* 2013; Wang *et al.* 2019). PacBio Iso-Seq has been used to study the transcriptomes of many organisms, often in the context of identifying splice variants, or alternative transcripts (Gordon *et al.* 2015; Rhoads and Au 2015; Xu *et al.* 2015; Abdel-Ghany *et al.* 2016; Guo *et al.* 2016; Wang *et al.* 2016; Weirather *et al.* 2017). Alternative transcripts can be identified using CCS because this technology obtains consensus sequences for full-length single transcripts (Zhao *et al.* 2019). In the same way, CCS can also be used to distinguish paralogs or gene duplicates.

To create an Iso-Seq library for *S. noctiflora*, the four RNA extractions (1.5 µg each) were pooled into a single sample and sent to the Arizona Genomics Institute for PacBio Iso-Seq library construction and sequencing. The library was constructed on the pooled RNA sample using Poly(A) selection, following the standard PacBio Iso-Seq protocol (“Procedure & Checklist—Iso-Seq Template Preparation for Sequel Systems,” Pacific Biosciences, PN-101-070-200 Version 06, September 2018), and then was sequenced with a PacBio Sequel (first generation) platform on two SMRT Cells.

Raw movie files of long-read, single-molecule sequences (one per SMRT Cell) were processed using the PacBio Iso-Seq v3.1 pipeline (Anvar *et al.* 2018; https://www.pacb.com/products-and-services/analytical-software/rna-sequencing/). Circular consensus sequence calling was performed on each movie file separately using the command *ccs* with the recommended parameters *–noPolish* and *–minPasses 1*. Next, primer removal was performed on each dataset by running the command *lima* with parameters *–isoseq* and *–no-pbi*. Poly(A) tails were trimmed and concatemers were removed using the *refine* command with the parameter *–require-polya*. Data from the two cells were merged at this point using the commands *dataset create –type TranscriptSet* and *dataset create –type SubreadSet*. Finally, the merged data were run through the *cluster* and *polish* commands. We also ran the *cluster* and *polish* commands on each dataset individually after skipping the merge step.

Trinotate v3.2.0 (Bryant *et al.* 2017) was used to annotate the final polished sequences produced by the Iso-Seq pipeline after merging the datasets. To complete this process, we used Transdecoder v5.5.0 (https://github.com/TransDecoder/TransDecoder/wiki), SQLite v3 (Kreibich 2010), NCBI BLAST + v2.2.29 (Camacho *et al.* 2009), HMMER v3.2.1 (including RNAMMER) (Lagesen *et al.* 2007; Potter *et al.* 2018), signalP v4 (Petersen *et al.* 2011), and tmhmm v2 (Krogh *et al.* 2001). The Pfam (Bateman *et al.* 2004) and UniProt (UniProt Consortium 2015) databases were included in the Trinotate installation. The transcripts and Transdecoder-predicted peptides were searched against the respective databases, following the standard Trinotate pipeline. All of these results were loaded into a Trinotate SQLite database.

Cogent v4.0.0 (https://github.com/Magdoll/Cogent/wiki) and minimap2 v2.17 (Li 2018) were used to conduct family findings on the final sequences by the Iso-Seq pipeline by partitioning sequences into groups based on similarity. While the Iso-Seq pipeline collapses reads into individual transcripts, it does not collapse alternative transcripts originating from the same gene. Cogent further collapses alternative transcripts into groups, where each group is meant to represent a single gene. Next, coding genome reconstruction was performed on each group from the above step; thus, the Cogent output included both a file containing groups of alternative transcripts (final.partition.txt at https://github.com/alissawilliams/Silene_noctiflora_IsoSeq) and a transcript-based genome. Finally, this transcript-based genome was used to determine total gene and isoform (alternative transcript) counts via cDNA_Cupcake scripts (https://github.com/Magdoll/cDNA_Cupcake/wiki; Jeffries *et al.* 2020; Wang *et al.* 2020). A modified form of the script *make_file_for_sampling_from_collapsed.py* was run with the parameter *–include_single_exons* in order to include all transcripts in the analysis. Gene and isoform counts were calculated using custom Python and R scripts on the resultant file. These Cogent, minimap2, and cDNA_Cupcake steps were performed on the merged dataset as well as individually on the datasets from each SMRT Cell.

We used genes from the plastid caseinolytic protease (Clp) as a case study to assess the ability of Iso-Seq dataset to distinguish paralogs (gene duplicates) of various levels of divergence. To identify nuclear-encoded plastid Clp core genes in our dataset, we used blastn in conjunction with the Cogent output. There are eight nuclear-encoded plastid Clp core genes in *Arabidopsis thaliana*: *CLPP3-6* and *CLPR1-4* (Nishimura and van Wijk 2015). In addition, the genus *Silene* shares a duplication of *CLPP5*, denoted *CLPP5A* and *CLPP5B* (Rockenbach *et al.* 2016). We obtained the sequences of all nine of these genes from a previous study (Rockenbach *et al.* 2016) and used them as queries in blastn searches against the *S. noctiflora* Iso-Seq transcriptome. We then identified which groups of collapsed alternative transcripts (from the Cogent output) contained these BLAST hits. BLAST hits for eight of the nine nuclear-encoded Clp core subunits in *Silene* (including *CLPP5A* and *CLPP5B*) were found in a single Cogent group. The sequences within each group were confirmed to represent a single gene via alignment and manual inspection; thus, these eight core subunits are single copy in *S. noctiflora*. However, in the case of *CLPR2*, two different Cogent groups contained relevant transcripts, indicating a possible case of gene duplication. Sequence alignment and manual inspection of the transcripts within these two Cogent groups revealed that one group contained two unique sequences. These data, along with sequencing results from a separate project in which we cloned two versions of *S. noctiflora CLPR2* using primers designed for *S. latifolia CLPR2*, suggested that there are actually three distinct *CLPR2* sequences in *S. noctiflora*. In the subsequent phylogenetic analysis of *CLPR2*, we used the longest sequences from each of the three identified groups.

A phylogenetic tree was constructed using sequences from the three different *S. noctiflora CLPR2* genes. In addition to the three *S. noctiflora* sequences, we also included *Agrostemma githago*, *S. conica, S. latifolia*, *Silene paradoxa*, and *Silene vulgaris CLPR2* sequences from a previous study (Rockenbach *et al.* 2016), as well as three *S. undulata CLPR2* sequences identified using blastn against the *S. undulata* TSA database (accession GEYX00000000). All 11 sequences were aligned using the *einsi* option in MAFFT v7.222 (Katoh and Standley 2013), and trimmed at the 5′ end based on the trimming conducted in Rockenbach *et al.* (2016). The resultant sequence file was run through jModelTest v2.1.10 (Darriba *et al.* 2012) to choose a model of sequence evolution. We chose the top model based on the Bayesian Information Criterion (K80+I) and ran PhyML v3.3 (Guindon *et al.* 2010) with 1000 bootstrap replicates and 100 random starts.

### Genome size estimates by flow cytometry

Leaf or seedling samples were collected from multiple individuals of varying age (between 2 and 14 weeks) for each of our target *Silene* species and shipped fresh to Plant Cytometry Services (Schijndel, Netherlands). Genome sizes were determined using the CyStain PI Absolute P reagent kit (05-5502). Samples were chopped with a razor blade in 500 μl of ice-cold Extraction Buffer in a plastic petri dish, along with *Pachysandra terminalis* tissue as an internal standard (3.5 pg/2C). After 30–60 seconds of incubation, 2 ml of Staining Buffer was added. Each sample was then passed through a nylon filter of 50 μm mesh size, and then incubated for 30+ min at room temperature. The filtered solution was then sent through a CyFlow ML flow cytometer (Partec GmbH). The fluorescence of the stained nuclei, which passed through the focus of a light beam with a 50 mW, 532 nm green laser, was measured by a photomultiplier and converted into voltage pulses. The voltage pulses were processed using Flomax version 2.4d (Partec) to yield integral and peak signals. Genome sizes were reported in units of pg/2C. The conversion used to report each size (x) in units of Gb was (x/2)*0.978 (Gregory *et al.* 2007).

### Karyotyping

*Silene noctiflora* OPL seeds were germinated on wet filter paper and grown for 5 days. Radicles were trimmed off and transferred to ice water for 24 hours. The radicles were then fixed in a 3:1 solution of absolute ethanol and glacial acetic acid and stored at −20°C. Chromosomes were visualized using a squash preparation with Feulgen staining. Fixed radicles were rinsed in distilled water for 5 minutes at 20°C. Radicles were then hydrolyzed in 5 M HCl at 20°C for 60 minutes followed by three rinses in distilled water. The hydrolyzed radicles were transferred to Schiff’s reagent to stain the DNA for 120 minutes at 20°C and were then destained by rinsing in SO_2_ water at 20°C three times for 2 minutes, two times for 10 minutes, once for 20 minutes, and then transferred to distilled water. Squashes were prepared by placing a piece of tissue in 45% acetic acid for 10 minutes and then minced on glass. A coverslip was placed over the minced tissue and pressed with enough pressure to produce a monolayer of nuclei. Slides were placed on dry ice for 1 minute, and the coverslip was removed. The slides were transferred to 96% ethanol for 2 minutes, air dried, and mounted with a mounting medium. Chromosomes were observed using a compound light microscope at 100× magnification.

### Genome sequencing and assembly

Extracted *S. noctiflora* OPL DNA samples were used for Illumina library construction and sequencing. A paired-end library with a target insert size of 275-bp was constructed at the Yale Center for Genome Analysis and sequenced on a 2 × 150-bp HiSeq 2500 run (three lanes). Two mate-pair libraries (with target insert sizes of 3–5 and 8–11 kb) were generated at GeneWiz and sequenced on a 2 × 150-bp HiSeq 2500 run (one lane each). Approximately 480, 250, and 230 M read pairs were generated for the 275, 3–5 kb, and 8–11 kb libraries, respectively. These reads are available via the NCBI SRA (accessions SRR9591157-SRR9591159). Reads were trimmed for quality and to remove 3′ adapters, using cutadapt v1.3 (Martin 2011) under the following parameters*: -n 3 -O 6 -q 20 -m 30 -a AGATCGGAAGAGCACACGTCTGAACTCCAGTCAC –paired-output*. The trimmed reads were assembled with ALLPATHS-LG release 44837 (Gnerre *et al.* 2011). Estimates of mean insert size and standard deviation for each library were provided as input for the assembly by first mapping a sample of reads to the published *S. noctiflora* plastid genome (GenBank accession JF715056.1). These estimates were as follows: 274 bp (±22 bp), 3752 bp (±419 bp), and 9873 bp (±1283 bp).

### BUSCO analyses

Benchmarking Universal Single-Copy Orthologs (BUSCO) analysis (Seppey *et al.* 2019) compares an assembly (transcriptomic or genomic) to a set of highly conserved orthologs from a particular clade in order to assess the completeness of the assembly. BUSCO (v4.1.4) analysis was performed on the Iso-Seq transcriptome and the genome assembly, as well as the output of the individual SMRT Cells. In each case, fasta files containing all genomic or transcriptomic sequences were run through BUSCO using the lineage eudicots_odb10 (2020-09-10) and default parameters. The graphical summary of results was produced using the script generate_plot.py included in the BUSCO installation.

### Data availability

The original subread bam files and final transcript sequences longer than 199 bp from the PacBio Iso-Seq transcriptome are available at NCBI Sequence Read Archive (SRA accession SRR11784995) and NCBI Transcriptome Shotgun Assembly Sequence Database (TSA accession GIOF01000000), respectively. The genome assembly has been deposited in GenBank (accession VHZZ00000000.1). Additional data have been provided at GitHub (https://github.com/alissawilliams/Silene_noctiflora_IsoSeq): (1) the full transcriptome as outputted by the PacBio Iso-Seq pipeline, (2) the annotation report for the transcriptome, (3) a custom script used to create a gene_trans_map file for our data in order to use Trinotate on non-Trinity-derived data (*i.e.*, transcripts derived from sources other than a Trinity assembly, in this case, Iso-Seq transcripts), (4) the Cogent output containing collapsed groups of transcripts, and (5) the set of trimmed, aligned sequences used in the *CLPR2* phylogenetic analysis.

## Results and discussion

### *Silene noctiflora* Iso-Seq transcriptome: gene content and duplication

Sequencing of the Iso-Seq library on two Sequel SMRT Cells produced 711,625 and 686,576 reads for the first and second cells, respectively, where each read was derived from a single molecule. The two SMRT Cells differed substantially in data yield, with totals of 12,765,109 and 21,844,543 subreads, corresponding to subread counts of 17.9 and 31.8 per read, respectively. These reads were merged into 65,642 distinct high-quality transcripts according to the thresholds of the Iso-Seq 3.1 *merge* and *polish* commands. Of these transcripts, only 14 were found to be nonplant sequences, all of which were derived from *Frankliniella occidentalis* (the western flower thrip), a common greenhouse pest that likely contaminated our tissue samples. We annotated these transcripts using Trinotate (Bryant *et al.* 2017); our dataset contains 69,846 total entries for the 65,642 transcripts (transcripts with multiple predicted proteins are represented by multiple entries). Of the 69,846 entries, 48,742 (74.3%) have an annotated PFAM domain, 47,504 (68.0%) have a KEGG annotation, and 55,993 (80.2%) have at least one predicted Gene Ontology term.

Each high-quality transcript represents collapsed reads, meaning that identical or nearly identical sequences are represented by the same final sequence. However, the Iso-Seq pipeline does not collapse alternatively spliced transcripts, or isoforms; thus, this final dataset includes multiple transcripts derived from the same genes. In addition to separately representing isoforms, the transcriptome data could also contain alleles of the same gene and transcripts from paralogs (gene duplicates). Given sufficiently divergent alleles or paralogs, pairs of these types of sequences will also be represented by separate final transcripts in this dataset. Due to the low levels of polymorphism and heterozygosity in *S. noctiflora* (Sloan *et al.* 2012a), we did not expect different alleles to comprise a major portion of this dataset.

**Figure 2:**
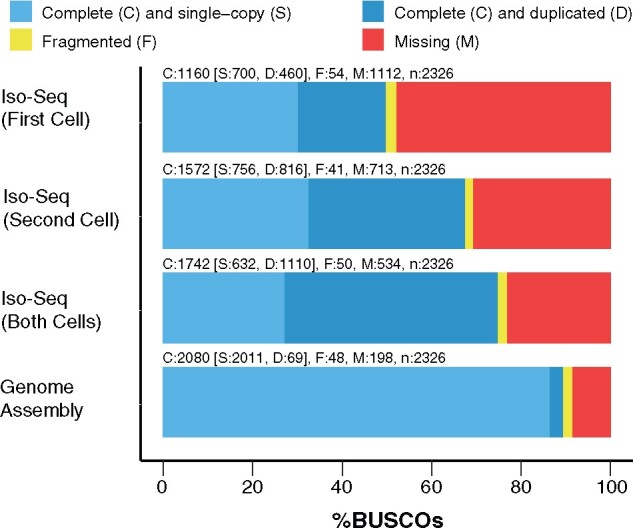
BUSCO analysis of the *S. noctiflora* genome assembly, Iso-Seq transcriptome (full dataset), and the individual SMRT Cells that were merged to create the Iso-Seq transcriptome.

Based on a BUSCO analysis (Seppey *et al.* 2019), the Iso-Seq transcriptome had a completeness of 74.9%. This estimate included a large number of duplicated BUSCOs (47.7%), but these do not necessarily represent true gene duplications for the reasons stated above (Figure 2). The merged dataset had a higher completeness percentage than either of the individual SMRT Cells, where the second SMRT Cell was more complete than the first, consistent with the differential data yield between the two cells (Figure 2). The estimated BUSCO completeness of the transcriptome was lower than that of the assembled nuclear genome (see below), which suggests that some genes with low or tissue-specific expression were not captured. Future efforts to generate deeper sequencing across a wider sample of tissues and environments may be beneficial in this respect.

We used the Cogent (https://github.com/Magdoll/Cogent/wiki) family finding algorithm to further collapse the transcripts into groups of isoforms (alternative transcripts) originating from the same gene. Notably, if paralogs (gene duplicates) have high enough sequence similarity, this binning could include them in the same group. We then used the Cogent data along with Cupcake (https://github.com/Magdoll/cDNA_Cupcake/wiki) to calculate the number of genes and isoforms represented in the transcriptome. Based on this analysis, the Iso-Seq transcriptome contains 14,126 *S. noctiflora* genes and 25,317 isoforms. Of the 14,126 genes, 7,027 had a single isoform (49.7%). We also calculated gene and isoform counts for each individual SMRT Cell; the first SMRT Cell produced 6,790 genes and 10,568 isoforms, while the second SMRT Cell produced 10,283 genes and 17,000 isoforms.

We wanted to test the ability of Iso-Seq to detect and distinguish known paralogs of varying levels of divergence using the Cogent family finding output. To this end, we used a sample gene family—the core subunit genes of the plastid Clp complex, as they have a rich history of paralogy. In *Escherichia coli* and most other bacteria, the core of the Clp complex, which is responsible for proteolysis, contains 14 identical subunits (Yu and Houry 2007). In cyanobacteria, gene duplication has led to four different core subunit-encoding genes (Stanne *et al.* 2007). Continued gene duplication in the land plant lineage has further reshaped this complex in plastids; the 14 core subunits are encoded by nine different genes in *A. thaliana*, eight of which are nuclear encoded (*CLPP3-6* and *CLPR1-4*), and one of which is plastid encoded (*clpP1*) (Nishimura and van Wijk 2015). Further, we had previously identified a more recent duplication of *CLPP5* in *Silene*, as well as duplications of the plastid-encoded *clpP1* in a small number of angiosperm species (Erixon and Oxelman 2008; Rockenbach *et al.* 2016; Williams *et al.* 2019). The Clp complex is one of the most highly expressed stromal proteases (Nishimura and van Wijk 2015). It is expressed in most tissues throughout the life stages of the plant, including the tissues from which we extracted RNA (Zheng *et al.* 2002). Thus, we would expect a transcriptome generated from the tissues we used to yield sequences of the various components of the Clp complex.

**Figure 3:**
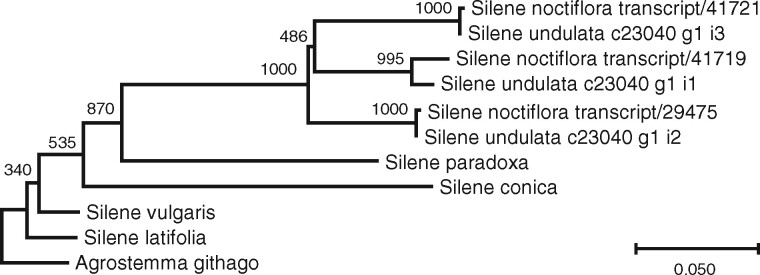
Phylogenetic analysis of *CLPR2* genes in *S. noctiflora* and related species. Branch lengths represent nucleotide sequence divergence. This tree was rooted on the *Agrostemma githago* sequence. The placement of *S. paradoxa* is in conflict with the species tree (Jafari *et al.* 2020), likely due to long branch attraction and the multiple independent evolutionary rate accelerations in this protein across *Silene* (Rockenbach *et al.* 2016).

We used the Cogent output to examine the nine nuclear-encoded Clp core genes in *S. noctiflora*. The core genes *CLPP3, CLPP4, CLPP5A, CLPP5B, CLPP6, CLPR1, CLPR3*, and *CLPR4* were each represented by a single group in the Cogent output, whereas *CLPR2* was represented by two groups. Upon further examination, one of these groups actually represented two different genes, yielding a total of three *CLPR2* genes in *S. noctiflora*. Thus, *CLPR2* was duplicated in this lineage, and then one paralog underwent a second gene duplication. Based on a phylogenetic analysis (Figure 3), these two duplications are shared with *Silene undulata* but none of the other sampled *Silene* species. Thus, these duplications likely occurred after *Silene* section Elisanthe (including *S. noctiflora*, *S. undulata*, and *Silene turkestanica*) diverged from the other members of the genus (Jafari *et al.* 2020; Moiloa *et al.* 2021).

The Iso-Seq data allowed us to identify transcripts from every known nuclear-encoded Clp core gene in *S. noctiflora*, including the closely related *CLPP5A* and *CLPP5B* subunits, as well as an additional, previously unreported triplication of *CLPR2*. To corroborate the triplication of *CLPR2* in *S. noctiflora* that was identified using the Iso-Seq transcriptome, we used the *CLPR2* sequence from Rockenbach *et al* (2016) as a query in a blastn search against the *S. noctiflora* genome assembly. This search returned four scaffold hits. Upon examination, each *CLPR2* gene identified in the Iso-Seq transcriptome was represented by one scaffold. The fourth scaffold represented all three gene copies in a short region of high sequence identity between them, suggesting collapsing of similar sequence content within the genome assembly. Thus, each *CLPR2* gene was fully represented by sequences on two scaffolds—there was one unique scaffold per gene containing most of the sequence and one scaffold containing sequence shared by all three genes.

### *Silene* genome size estimates and chromosome number

Genome sizes of *S. noctiflora, S. conica, S. vulgaris*, and *S. latifolia* were determined using flow cytometry. Our estimates for *S. vulgaris* and *S. latifolia* (1.07 and 2.67 Gb, respectively; Table 1) were concordant with previously published estimates for these two species of 1.11 and 2.64 Gb (Costich *et al.* 1991; Siroký *et al.* 2001). Interestingly, despite their similar and extreme patterns of organelle evolution (Sloan *et al.* 2012a, 2014), including large mitochondrial genomes, *S. noctiflora* and *S. conica* have very different nuclear genome sizes. We found their respective genome sizes to be approximately 2.74 and 0.93 Gb, respectively (Table 1), which are on opposite ends of the spectrum for *Silene* diploids (Pellicer and Leitch 2020). The *S. noctiflora* nuclear genome is almost threefold larger than that of *S. conica*, suggesting that mitochondrial genome size is not necessarily correlated with nuclear genome size.

**Figure 4:**
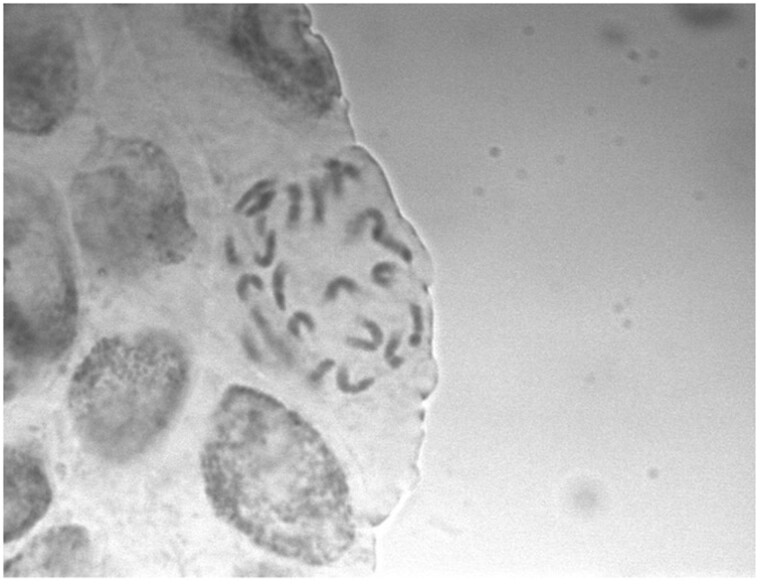
Micrograph verifying the diploidy of *S. noctiflora* at 100× magnification. Although an exact chromosome count is difficult to determine, this image suggests that *S. noctiflora* is a diploid with the typical number of 24 chromosomes previously documented in this species and the genus in general, rather than polyploid with 48 or more chromosomes (Bari 1973; McNeill 1980; Yildiz *et al.* 2008; Kemal *et al.* 2009; Gholipour and Sheidai 2010; Ghasemi *et al.* 2015; Mirzadeh Vaghefi and Jalili 2019).

Most diploids in the genus, including *S. noctiflora*, have a chromosome number of 2*n* = 24, which is likely the ancestral number (Bari 1973; McNeill 1980; Yildiz *et al.* 2008; Kemal *et al.* 2009; Gholipour and Sheidai 2010; Ghasemi *et al.* 2015; Mirzadeh Vaghefi and Jalili 2019). There are also numerous polyploid *Silene* species, including tetraploid, hexaploid, and octaploid forms (Kruckeberg 1960; Popp and Oxelman 2001, 2007; Popp *et al.* 2005; Bai *et al.* 2012). *Silene noctiflora* has been previously reported as a diploid (McNeill 1980; Yildiz *et al.* 2008; Ghasemi *et al.* 2015). Given its relatively large genome size, we sought to confirm this result in our sampled population with a karyotype analysis (Figure 4), which indeed supported the conclusion that *S. noctiflora* OPL is diploid.

### The *Silene noctiflora* nuclear genome

Illumina sequencing produced ∼50× coverage of the *S. noctiflora* genome for a 275-bp paired-end library and ∼15–20× for each of two mate-pair libraries. By performing a *de novo* assembly of these reads, we obtained a total assembly length (including estimated scaffold gaps) of 2.58 Gb, which is generally consistent with our estimate based on flow cytometry for *S. noctiflora* OPL (2.71 Gb). Given that we relied entirely on short-read sequencing technology, it was not surprising that the resulting assembly of this large genome was highly fragmented (79,768 scaffolds with a scaffold N50 of 59 kb; 222,040 contigs [minimum length of 1 kb for reporting contigs] with a contig N50 of 4.8 kb). Moreover, assembly gaps made up 73% of the total scaffold length, presumably representing the highly repetitive content that is typical of plant nuclear genomes. As such, the assembled gap-free sequences amount to only about a quarter of the genome (702 Mb). Given the expected low levels of polymorphism and heterozygosity in *S. noctiflora* (Sloan *et al.* 2012a), the assembly was interpreted as a single haplotype and no attempt was made to phase the two distinct haplotypes within the diploid.

BUSCO analysis (Seppey *et al.* 2019) provided an estimate of 89.5% completeness for the *S. noctiflora* genome assembly (Figure 2). Only 3.0% of BUSCOs were reported to be duplicated, in great contrast to the transcriptome, where 47.7% of BUSCOs were duplicated. Given that the final Iso-Seq dataset includes alternatively spliced transcripts as separate entries, it is not surprising that the transcriptome had a higher percentage of duplicated BUSCOs than the genome assembly.

As a complement to the Iso-Seq transcriptome, this *S. noctiflora* genome assembly should provide a useful resource to query for sequences of interest, especially in genic regions, and to compare against *S. latifolia* and other members of this genus. However, a more complete assembly that includes repetitive regions of the genome will require additional data from long-read technologies such as PacBio or nanopore sequencing. The Iso-Seq data generated in this study may be helpful in combination with improved genomic sequencing data in the future, as a means to improve scaffolding (Zhu *et al.* 2018), resolve paralogs (*e.g.*, the collapsed regions of the *CLPR2* paralogs in the genome assembly), and annotate gene models.
